# Beneficial and Pathogenic Arabidopsis Root-Interacting Fungi Differently Affect Auxin Levels and Responsive Genes During Early Infection

**DOI:** 10.3389/fmicb.2019.00380

**Published:** 2019-03-12

**Authors:** Anja K. Meents, Alexandra C. U. Furch, Marília Almeida-Trapp, Sedef Özyürek, Sandra S. Scholz, Alexander Kirbis, Teresa Lenser, Günter Theißen, Veit Grabe, Bill Hansson, Axel Mithöfer, Ralf Oelmüller

**Affiliations:** ^1^Department of Bioorganic Chemistry, Max Planck Institute for Chemical Ecology, Jena, Germany; ^2^Department of Plant Physiology, Matthias Schleiden Institute of Genetics, Bioinformatics and Molecular Botany, Friedrich-Schiller-University Jena, Jena, Germany; ^3^Research Group Plant Defense Physiology, Max Planck Institute for Chemical Ecology, Jena, Germany; ^4^Department of Genetics, Matthias Schleiden Institute of Genetics, Bioinformatics and Molecular Botany, Friedrich-Schiller-University Jena, Jena, Germany; ^5^Department of Evolutionary Neuroethology, Max Planck Institute for Chemical Ecology, Jena, Germany

**Keywords:** auxin, phytohormones, plant-fungus interaction, *Piriformospora indica*, *Mortierella hyalina*, *Alternaria brassicicola*, *Verticillium dahliae*, light sheet fluorescence microscopy

## Abstract

Auxin (indole-3-acetic acid, IAA) is an important phytohormone involved in root growth and development. Root-interacting beneficial and pathogenic fungi utilize auxin and its target genes to manipulate the performance of their hosts for their own needs. In order to follow and visualize auxin effects in fungi-colonized Arabidopsis roots, we used the dual auxin reporter construct *DR5*::*EGFP-DR5v2*::*tdTomato* and fluorescence microscopy as well as LC-MS-based phytohormone analyses. We demonstrate that the beneficial endophytic fungi *Piriformospora indica* and *Mortierella hyalina* produce and accumulate IAA in their mycelia, in contrast to the phytopathogenic biotrophic fungus *Verticillium dahliae* and the necrotrophic fungus *Alternaria brassicicola*. Within 3 h after exposure of Arabidopsis roots to the pathogens, the signals of the auxin-responsive reporter genes disappeared. When exposed to *P. indica*, significantly higher auxin levels and stimulated expression of auxin-responsive reporter genes were detected both in lateral root primordia and the root elongation zone within 1 day. Elevated auxin levels were also present in the *M. hyalina*/Arabidopsis root interaction, but no downstream effects on auxin-responsive reporter genes were observed. However, the jasmonate level was strongly increased in the colonized roots. We propose that the lack of stimulated root growth upon infection with *M. hyalina* is not caused by the absence of auxin, but an inhibitory effect mediated by high jasmonate content.

## Introduction

Auxin plays a central role for root growth and participates in many aspects of root development, including cell elongation, differentiation (Rahman et al., 2007), lateral root, and root hair formation (Masucci and Schiefelbein, 1994, 1996; Pitts et al., 1998; Reed et al., 1998; Casimiro et al., 2001; Bhalerao et al., 2002), and gravitropic responses (Rashotte et al., 2000; Sukumar et al., 2009). Auxin action depends on its differential distribution within plant tissues, where it forms local maxima or gradients between cells. The different auxin levels arise from auxin metabolism (biosynthesis, degradation, and conjugation), long-distance transport and directional cell-to-cell translocation (Petrášek and Friml, 2009). Auxin interacts with other phytohormones and imbalances in the phytohormone levels have severe consequences. Well-studied examples are the auxin/cytokinin balance (De Rybel et al., 2014) and the control of lateral root growth by an interplay between auxin, abscisic acid, brassinosteroids, ethylene, and jasmonates (cf. Fukaki and Tasaka, 2009; Ishimaru et al., 2018). In addition, auxin produced by plant-associated microorganisms mediates phytostimulatory effects on plants. Pathogens can manipulate auxin biosynthesis, signaling, and transport pathways to promote host susceptibility. Auxin responses are also coupled to antagonistic and synergistic interactions with salicylic acid (SA)- and jasmonic acid (JA)-mediated defenses, respectively (Hoffmann et al., 2011; Naseem et al., 2015; Huang et al., 2017). Jasmonates participate in the regulation of primary root growth and reproductive development, thereby interacting with auxins. Crosstalk can occur at multiple levels, including hormone perception, since indole-acetic acid (IAA) and JA-isoleucine (JA-Ile) are perceived by SCF E3-ligases through the interaction of IAA- and JA-related regulators of gene expression and the modulation of each other's homeostasis (Hoffmann et al., 2011; Naseem et al., 2015). Auxin induces JA biosynthesis and JA controls the expression of some of the auxin biosynthetic genes (Dombrecht et al., 2007; Sun et al., 2009; Hentrich et al., 2013). Furthermore, high JA concentrations reduce the accumulation of the PIN-FORMED1 (PIN1) and PIN2 auxin transporters (Gutjahr et al., 2005; Hoffmann et al., 2011; Sun et al., 2011). IAA binds to the receptor AUXIN SIGNALING F-BOX PROTEIN TIR1 and inhibits a family of transcriptional repressors known as AUXIN/IAAs (Dharmasiri et al., 2005; Kepinski and Leyser, 2005; Salehin et al., 2015). In the presence of IAA, the SKP1-CULLIN1-F-BOX TIR1 ubiquitin ligase complex binds to AUXIN/IAAs and triggers their degradation (Calderon-Villalobos et al., 2010).

The vast majority of roots in the ecosystems is associated with beneficial fungi. They form mycorrhizal symbiosis or interact with endophytes. Plants benefit from these associations in many ways, such as better access to water and nutrients, promotion of growth and biomass production and resistance to biotic and abiotic stress. The fungi are supplied with reduced carbon from the host photosynthesis and live in a protected shelter. The beneficial symbiosis results in alterations of the host phytohormone levels. For example, beneficial microbes synthesize auxin or auxin precursors, interfere with the plant auxin biosynthesis and metabolism or manipulate auxin signaling and responses. In many cases, the microbes utilize the plant phytohormone machinery and reprogram it to their own needs (Xu et al., 2018, and references therein).

In this study, we used the previously described auxin-responsive reporter system *DR5*::*EGFP-DR5v2*::*tdTomato* (Ulmasov et al., 1997; Liao et al., 2015) to monitor how the root-interacting microbes *Piriformospora indica, Mortierella hyalina, Verticillium dahlia*, and *Alternaria brassicicola* manipulate the root auxin metabolism during early phases of the interaction with Arabidopsis roots. *P. indica*, a member of *Sebacinales*, grows inter- and intracellularly and forms pear shaped spores, which accumulate within the roots and on the root surface (Peškan-Berghöfer et al., 2004; Oelmüller et al., 2009; Camehl et al., 2011). The fungus promotes the growth of the host plants (Peškan-Berghöfer et al., 2004), induces early flowering (Pan et al., 2017) and confers resistance against abiotic and biotic stress (Narayan et al., 2017; Zhang et al., 2017; Vahabi et al., 2018). The endophyte produces indole derivatives but they are not required for growth promotion in barley roots (Hilbert et al., 2012). *P. indica* releases cellotriose that induces root-specific [Ca^2+^]_cyt_ elevation required for the activation of a mild defense response (Vadassery et al., 2008; Johnson et al., 2018a; Oelmüller, 2018). Root-specific [Ca^2+^]_cyt_ elevation is also induced by an exuded info-chemical from *M. hyalina* (Johnson et al., 2018b), a growth promoting fungus of *Mortierellales* (Shinmen et al., 1989) which results in defense gene activation. The fungus promotes growth of the aerial parts of Arabidopsis via a fungus-released volatile, while the growth behavior of colonized roots resembles that of un-colonized controls (Johnson et al., 2018b). *V. dahliae* is hemibiotroph with an initial biotrophic life phase in the root xylem, followed by a necrotrophic phase once the hyphae reach the aerial plant tissues. While infected roots show little or no disease symptom development during the biotrophic phase, the fungus blocks xylem transport and synthesizes a cocktail of toxins during the necrotrophic phase, which results in wilting and disease symptom development in the leaves of *Brassicaceae* species (Pemberton and Salmond, 2004; Gijzen and Nürnberger, 2006; Qutob et al., 2006; van der Does and Rep, 2007; Bolton et al., 2008; de Jonge and Thomma, 2009; van Esse et al., 2009; de Jonge et al., 2010, 2011; Oliva et al., 2010; Klosterman et al., 2011; Scholz et al., 2018). The necrotrophic fungus *Alternaria* causes black leaf spot disease in crucifers and was used in this study since it infects both leaves and roots. Root infection induces rapid [Ca^2+^]_cyt_ elevations which results in host defense gene activation, jasmonate accumulation, ROS production and innate immunity. A few days after root infection, the Arabidopsis seedlings are dead (Johnson et al., 2018b).

In this study, we addressed the hypothesis that interactions of Arabidopsis roots with pathogenic and beneficial fungi are accompanied by different activation of auxin-responsive genes during very early phases of contact. Our results suggest that the activation of auxin-responsive genes and auxin-induced developmental programs in the colonized roots are controlled by the phytohormone balance, rather than by the auxin concentration alone.

## Material and Methods

### Plant Material and Growth Conditions

Seedlings of *Arabidopsis thaliana* containing the dual auxin reporter construct *DR5::EGFP-DR5v2::tdTomato* were generally described in Ulmasov et al. (1997) and Liao et al. (2015). Here, plants containing T-DNA comprising construct *pGWB601-DR5::EGFP-DR5v2::tdTomato* were used, which had been generated as described (Kirbis, 2017). The seeds were surface-sterilized and placed on petri dishes containing full Murashige-Skoog nutrient medium (MS) (Murashige and Skoog, 1962) supplemented with 40 mM sucrose, 2.6 mM 2-(N-morpholino)ethanesulfonic acid and 1% Kobe Agar (all supplies from Carl Roth, Karlsruhe, Germany). After a 48 h stratification at 4°C, plates were incubated vertically for 10 days at 22°C under long day conditions (16 h light/ 8 h dark; 80 μmol m^−2^ s^−1^).

### Fungal Material and Growth Conditions

*P. indica* (syn. Serindipita indica) was grown on petri dishes with modified Kaefer's medium (KM) as previously described (Verma et al., 1998; Peškan-Berghöfer et al., 2004) and kept in the dark at room temperature for 3 weeks. *A. brassicicola, M. hyalina*, and *V. dahliae* were cultivated for 2 weeks at 22 ± 1°C on potato dextrose agar (PDA) medium as reported in Johnson et al. (2014). *A. brassicicola, M. hyalina*, and *V. dahliae* were obtained from the Jena Microbial Resource Center and *P. indica* was provided by Ajit Varma (Amity Institute of Microbial Technology, India).

Spore suspensions of the fungi were prepared according to Johnson et al. (2014) by rinsing the plates containing the fungi with sterile H_2_O, gently scraping the spores and hyphae off the plate followed by filtration through a sterilized nylon membrane (75 μm pore size). The spore concentration was determined using a hemocytometer and adjusted with sterile H_2_O containing 0.01 % Tween-20 to 2 × 10^6^ spores/ml. As control the same solution was used without spores. Viability of the spores was routinely checked via germination tests.

### Co-cultivation for Fluorescence Microscopy

**Figure 1:**
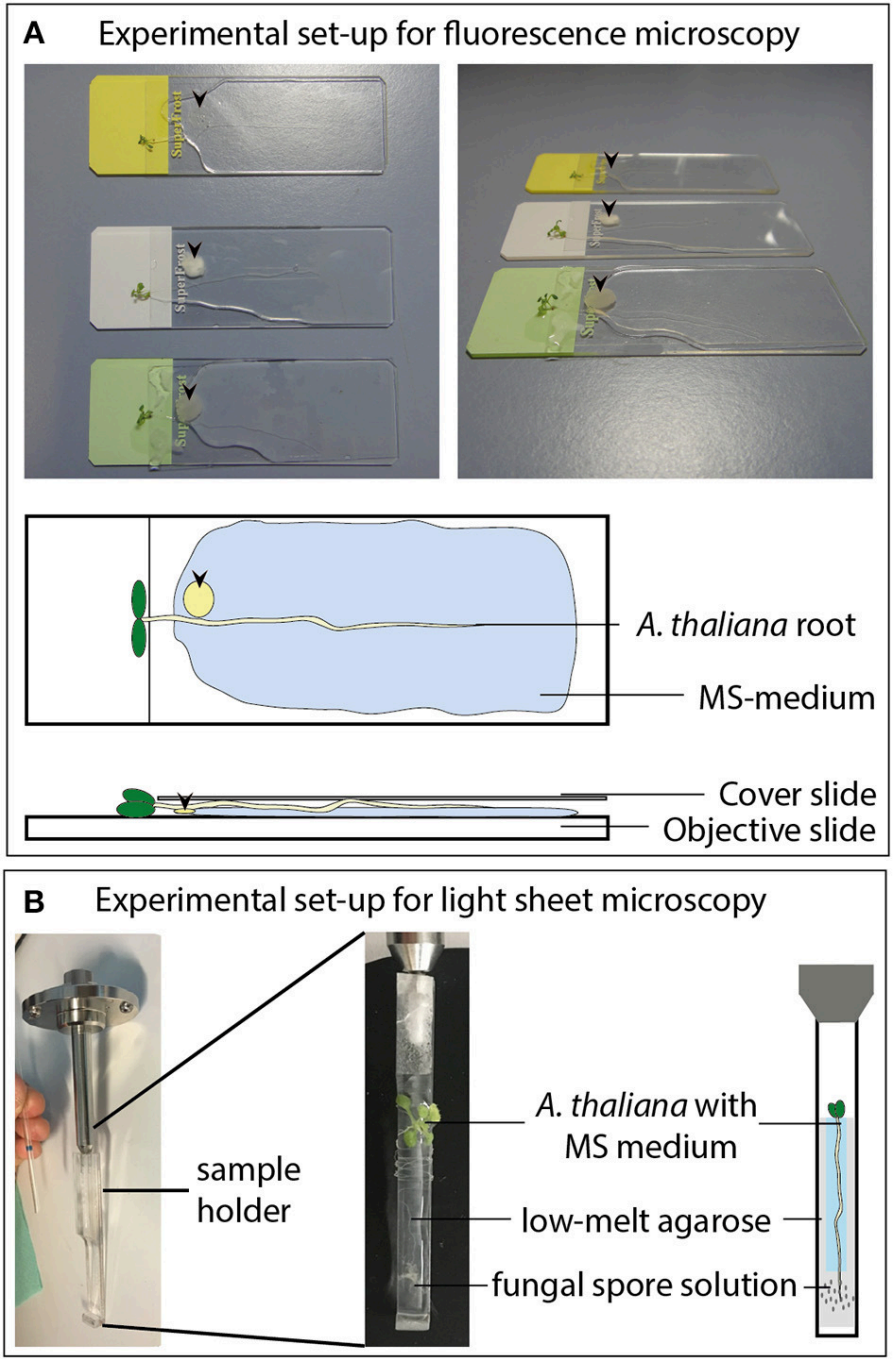
Experimental set-up for the fluorescence imaging measurements. **(A)** Co-cultivation of *A. thaliana* seedlings with different fungi on objective slides for fluorescence microscopy. Arrow heads indicate the position of the fungal plaques. **(B)** Co-cultivation of *A. thaliana* roots with fungal spore solutions (2 μl of 2 × 10^6^ spores per ml) for light sheet microscopy.

In order to investigate the impact of different fungi on the distribution of auxin maxima, Arabidopsis seedlings containing the reporter construct *pGWB601-DR5*::*EGFP-DR5v2*::*tdTomato* were co-cultivated with fungal plaques of *P. indica, A. brassicicola, M. hyalina*, or *V. dahliae* as described previously by Johnson et al. (2011, 2014) with modifications. For co-cultivation with subsequent fluorescence microscopy, 12 day-old plants were placed on microscope slides on top of a thin layer of full MS medium containing 1% Kobe agar and a plaque (5 mm diameter) of medium (PDA or KM, for control), or fungus agar cultures were applied (see Figure 1A). The seedling and fungal plug were coated with sterile tap water, covered with a cover slip and placed in a petri dish until microscopy was performed. The plates were then sealed with Parafilm (American National Can Company, Greenwich, USA) to prevent drying out of the sample and kept at 22°C under long day conditions (16 h light/8 h dark; 80 μmol m^−2^ s^−1^) for 1 to 3 days. The fluorescence of the reporter construct was analyzed by fluorescence microscopy (see below) after 24 h of incubation. To analyze the dose-dependent induction of fluorescence, the MS agar was supplemented with 0, 5, 10, or 20 μM of IAA (Merck, Darmstadt, Germany) or 0, 1, 5, or 10 μM of JA (Sigma-Aldrich, Taufkirchen, Germany).

### Fluorescence Microscopy

The entire Arabidopsis roots, elongation zones, root tips and primordia were imaged using an AXIO Imager.M2 (Zeiss Microscopy GmbH, Jena, Germany) equipped with a 10x objective (N-Achroplan 10x/0.3). The bright field and fluorescence images (EX 545/25 and EM 605/70) were recorded with a color camera (AXIOCAM 503 color Zeiss, Jena, Germany) by use of an EGFP (EM 525/50 nm) and DsRED filter (EM 605/70 nm). Digital images were processed with the ZEN software (Zeiss, Jena, Germany), treated with Adobe® PhotoShop to optimize brightness, contrast and coloring and to overlay the photomicrographs. The quantification of fluorescence was measured using the ZEN software by analyzing a region of interest at the root tip and/or the whole root.

### Light Sheet Fluorescence Microscopy

Twelve day-old Arabidopsis seedlings (grown as described above) were mounted on a custom plastic holder (see Figure 1B). Afterwards the root tip was infected with 2 μl of a *P. indica, A. brassicicola, M. hyalina*, or *V. dahliae* spore suspension (2 × 10^6^ spores/ml) or sterile water containing 0.01 % Tween-20 as a control. The whole plant was fixed using 2% low melting agarose (Carl Roth, Karlsruhe, Germany) and Parafilm to prevent unspecific movement (see Figure 1B). The measuring chamber was flooded with full Murashige-Skoog nutrient medium to ensure optimal nutrient supply during the whole measurement. Time series of the root response to the spore treatments were recorded on a LightSheet.Z1 (ZEISS, Oberkochen, Germany) equipped with a W Plan Apochromat 20x/1.0 UV-VIS (ZEISS, Oberkochen, Germany) and two lasers (laser lines: Argon 488 nm and Helium-Neon 561 nm). The red fluorescence was visualized using the 561 nm Helium-Neon laser at 20% transmission with a LBF 405/488/561 emission filter BP 575-615. For all recordings an exposure time of 350 ms was used at a zoom of 0.36 and a light sheet thickness of 6.68 μm. Image stacks series were acquired at 1,920·pixels, 20 μm z-thickness and 16 bit every 30 min for 24 h. Representative intensity kinetics were generated with the ZEN software (Zeiss, Jena, Germany).

### Co-cultivation for Phytohormone Analyses

For phytohormone analyses, 15 Arabidopsis seedlings were positioned on a full MS square plate on a sterilized nylon membrane, stratified for 2 days at 4°C and vertically grown for 10 days at 22°C under long day conditions (16 h light/8 h dark; 80 μmol m^−2^ s^−1^). In accordance with the previous plaque treatment, a 5 mm broad stripe of PDA medium with or without (control) fungal mycelium of *A. brassicicola, M. hyalina*, or *V. dahliae* was placed on the roots and incubated for 1 day. For co-cultivation with *P. indica*, a stripe of KM medium with or without fungus was used for treatment. The plates were co-cultured for 24 h before the entire root of each plate was collected, immediately frozen in liquid nitrogen and stored at −80°C before phytohormone extraction. Mycelium from each fungus grown alone was additionally collected from a whole plate, frozen and kept at −80°C.

### Quantification of Phytohormones

Prior to phytohormone extraction, the collected fungal samples (40–200 mg fresh weight, *n* = 3) were freeze-dried in a Modulyo®D Freeze Dryer (Thermo Scientific, Waltham, USA) for 48 h. The freeze-dried fungal samples and the collected root tissue of co-cultivated seedlings (1 sample = 12–15 seedlings from the same plate; which corresponded to 30–150 mg fresh weight, *n* = 8) were weighed and homogenized using a Geno/Grinder® (Spex SamplePrep, Stanmore, UK) at 1,100 rounds per min for 1 min. As described in Almeida Trapp et al. (2014), 1 ml of methanol: water (7:3) containing 20 μg/ml of d4-SA and d5-IAA as well as 10 μg/ml d5-JA and d6-ABA was added to the powdered root and fungal material. After mixing, samples were shaken for 30 min and centrifuged at 16,000 *g* at 4°C for 5 min. Subsequently the supernatant was transferred into a new tube and concentrated for 4 h at 45°C in an Eppendorf Concentrator plus (Eppendorf AG, Hamburg, Germany). The concentrate was resuspended in 100 μl of 50% methanol with 0.05% formic acid, mixed and centrifuged at 16,000 *g* at 4°C for 10 min. Afterwards the supernatant was collected and measured on an Agilent 1100 HPLC system (Agilent Technologies, Böblingen, Germany) connected to a LTQ Orbitrap mass spectrometer (Thermo Scientific, Waltham, USA) (Almeida Trapp et al., 2014).

### Statistics

Statistical analyzes of phytohormone data were performed in R studio (version 1.1.456), using one way ANOVA on log transformed data to ensure that the residues followed a normal distribution. Tukey's HSD test was used as *post-hoc* test to examine the differences between factor levels (treated plants vs. control) or multiple comparison for the amount of phytohormones present in fungi samples.

## Results

### The Reporter Construct *DR5*::*EGFP-DR5v2*::*tdTomato* Responds to Exogenously Applied IAA in a Concentration-Dependent Manner in Arabidopsis Roots

**Figure 2:**
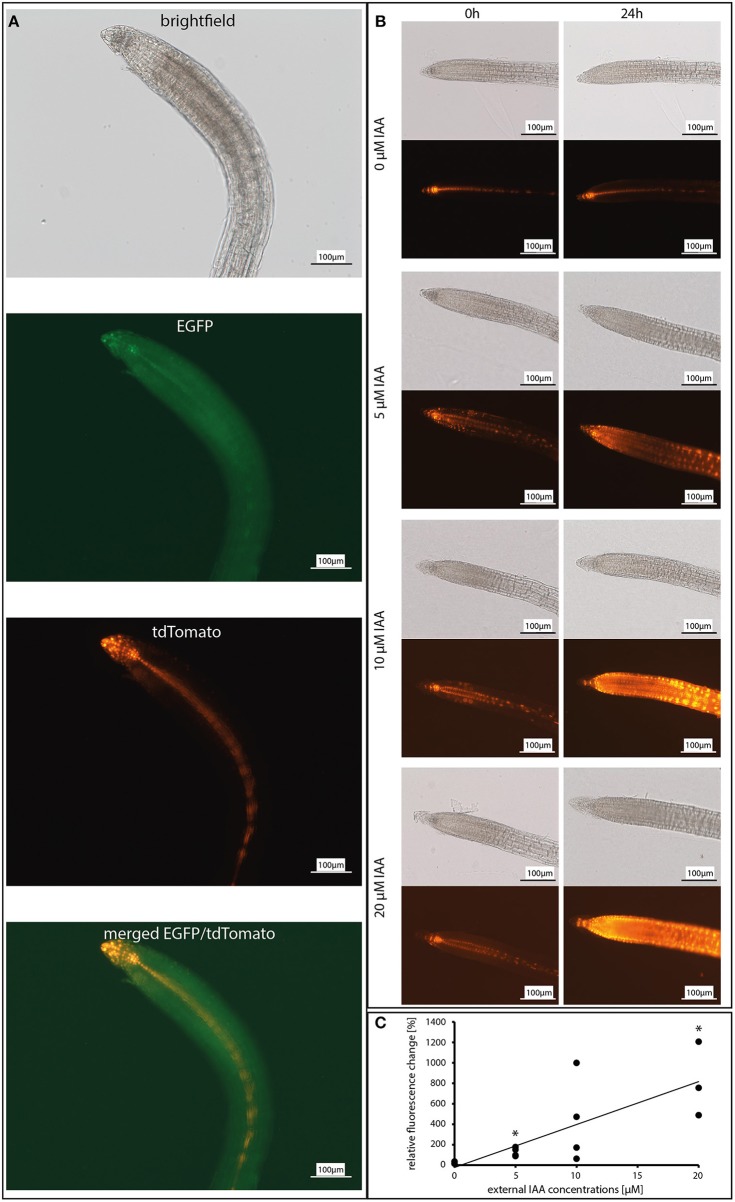
Dose-response curve of the reporter construct *DR5::EGFP-DR5v2::tdTomato* in response to incubation with 0, 5, 10, and 20 μM IAA (indole-3-acetic acid). **(A)** Co-localization of the *EGFP* and *tdTomato* expression in 12 day-old *A. thaliana* roots. Shown are the brightfield image, GFP channel (for EGFP), DS red channel (for tdTomato), and a merged image of the GFP and the DS red channel. **(B)** Fluorescence images of the tdTomato signal after 0 and 24 h. **(C)** Graphic presentation of the relative fluorescence change in response to exogenous IAA concentrations (*n* = 3–4). Asterisks indicate significant differences to 0 μM IAA (*p* < 0.05).

To test the expression of the auxin reporters to exogenously applied IAA, the roots of 12 day-old Arabidopsis seedlings were incubated with increasing IAA concentrations. After 24 h, the EGFP and tdTomato fluorescence was monitored (for experimental set-up see: Figures 1A, 2A,B). Without exogenously applied IAA, the fluorescence from both reporters was detectable in the quiescent center, columella, pericycle, and vascular system of the roots (Figure 2A, second image, EGFP; third image, tdTomato; fourth image, merged). Incubation with the lowest applied concentration of 5 μM IAA resulted in a significant increase in both fluorescence signals which were extended to the rhizodermal cell layers (Figure 2B). The intensity of the signals after the application of 5 μM IAA was twice as high as in the untreated controls and increased almost linear with increasing IAA concentrations (Figures 2B,C). Because the fluorescence signals were detectable throughout the entire root tissues (Figure 2B) exogenously applied IAA did not cause cell- or tissue-specific induction of the reporter genes. For these experiments and the pictures for the physiological experiments shown below, the fluorescence from both reporters was always measured; however, the overlay analysis never discovered meaningful differences (see Figure 2B). Since the fluorescence signal for the EGFP reporter was much lower than the one observed for the tdTomato reporter, the cell- and tissue-specific resolution was better for the latter reporter system. Therefore, for the presentation of the data, we show the results for the stronger fluorescent tdTomato reporter. Furthermore, the linear increase of the fluorescence signals with increasing exogenous applied IAA (Figure 2B) makes it likely that the fluorescence reports fungus-induced changes in the expression of auxin-responsive genes in the root tissues.

### Live Imaging of Root Infection Reveals Fungus-Specific Redistribution of Auxin Maxima in *A. thaliana*

**Figure 3:**
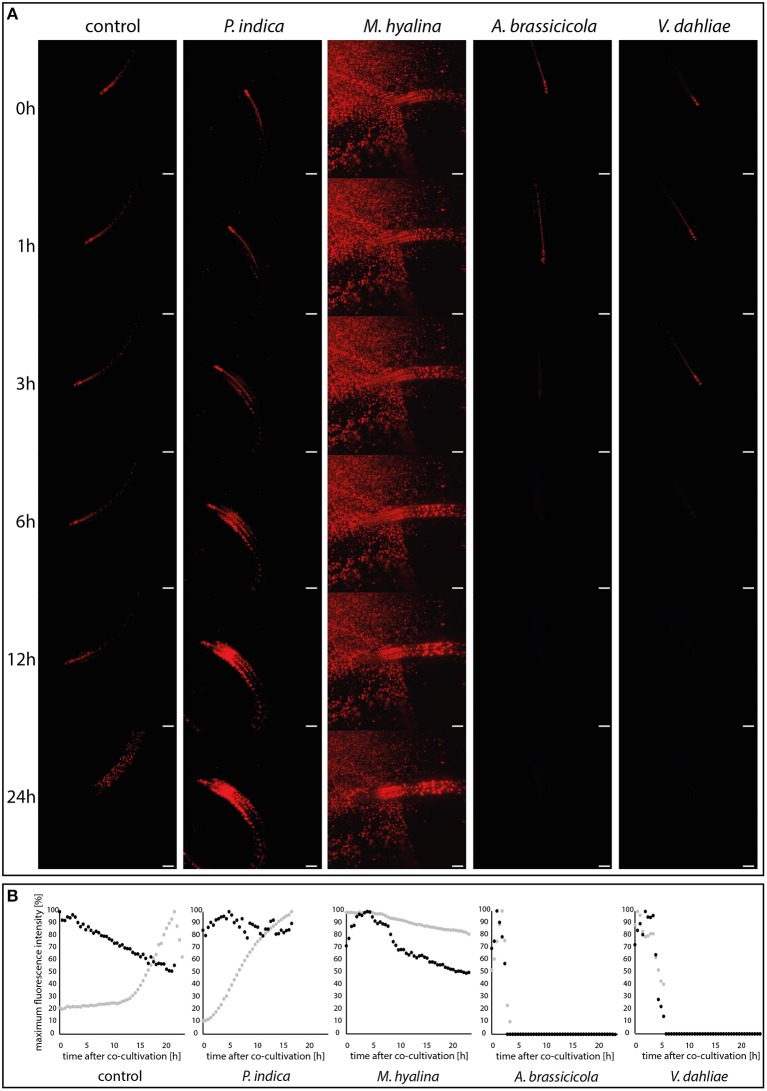
Visualization of auxin maxima using the reporter construct *DR5::EGFP-DR5v2::tdTomato* during plant-fungus interaction. **(A)** Light sheet images of 24 h-recordings of control and co-cultivation with spore solutions of *P. indica, M. hyalina, A. brassicicola*, and *V. dahliae*. Scale bar = 200 μm. Background fluorescence in *M. hyalina* co-culture is due to autofluorescence of spores. **(B)** Quantification curves of fluorescence maxima of each co-culture in a time-dependent manner. Black dots, root tip; gray dots, whole root (*n* = 3).

The auxin response was monitored over 24 h after spore application to the roots by measuring the fluorescence emitted from the DR5v2::tdTomato reporter using light sheet fluorescence microscopy (Figures 1B, 3A,B). Representative pictures from the movies (Supplementary Videos S1–S5) are shown in Figure 3A. Figure 3B shows quantified fluorescence signals of the entire roots and of the root tip separately. Without spores, the tdTomato-derived fluorescence displayed initially a stable signal in the quiescent center, columella, pericycle and vascular system (Figure 3A). After 10–12 h, the overall fluorescence in the entire root increased. In the root tip, we observed a continuous decrease of the fluorescence (see Figure 3B), while the fluorescence from cells located in the elongation zone and from rhizodermal cells increased. The measurements were stopped after 24 h because the reporter system showed the first bleaching symptoms at the root tip.

Application of spores from *P. indica* led to an almost linear increase of the fluorescence in the entire root which started after a lag phase of ~1 h. Most of the fluorescence was emitted from cells of the basal meristem and the transition zone. Furthermore, the decrease of the fluorescence emitted from the root tip, which was observed for uncolonized roots, was stopped. This clearly indicates that signals from *P. indica* activate the auxin-responsive reporter gene in Arabidopsis roots.

A quite different scenario was observed for *M. hyalina*. The overall emission from the entire root remained almost constant over the measuring period (< 20% decrease over the 24 h period), indicating that the fungus inhibits the stimulation which is observed for uncolonized and *P. indica*-colonized roots (more than 70% increase over the 24 h period). Furthermore, after initial stimulation of the fluorescence emission, the activity also declines at the root tip, similar to the decline observed for the uncolonized control. These results demonstrate that *M. hyalina* prevents the activation of the reporter gene.

The two pathogenic fungi *A. brassicicola* and *V. dahliae* showed almost identical effects on the expression of the reporter gene, although the first fungus is a necrotroph and the second a biotroph. A few hours after spore application the fluorescence in the roots and root tips completely disappeared. Thus, signals from the fungi very likely inhibit the activation of the reporter gene.

In summary, compared to uncolonized roots, *P. indica* stimulated and the pathogens inhibited the expression of the reporter gene. Besides an initial positive effect on the root tip, the reporter gene activity is not altered by *M. hyalina*.

### Phytohormone Levels in Colonized *A. thaliana* Roots

**Figure 4:**
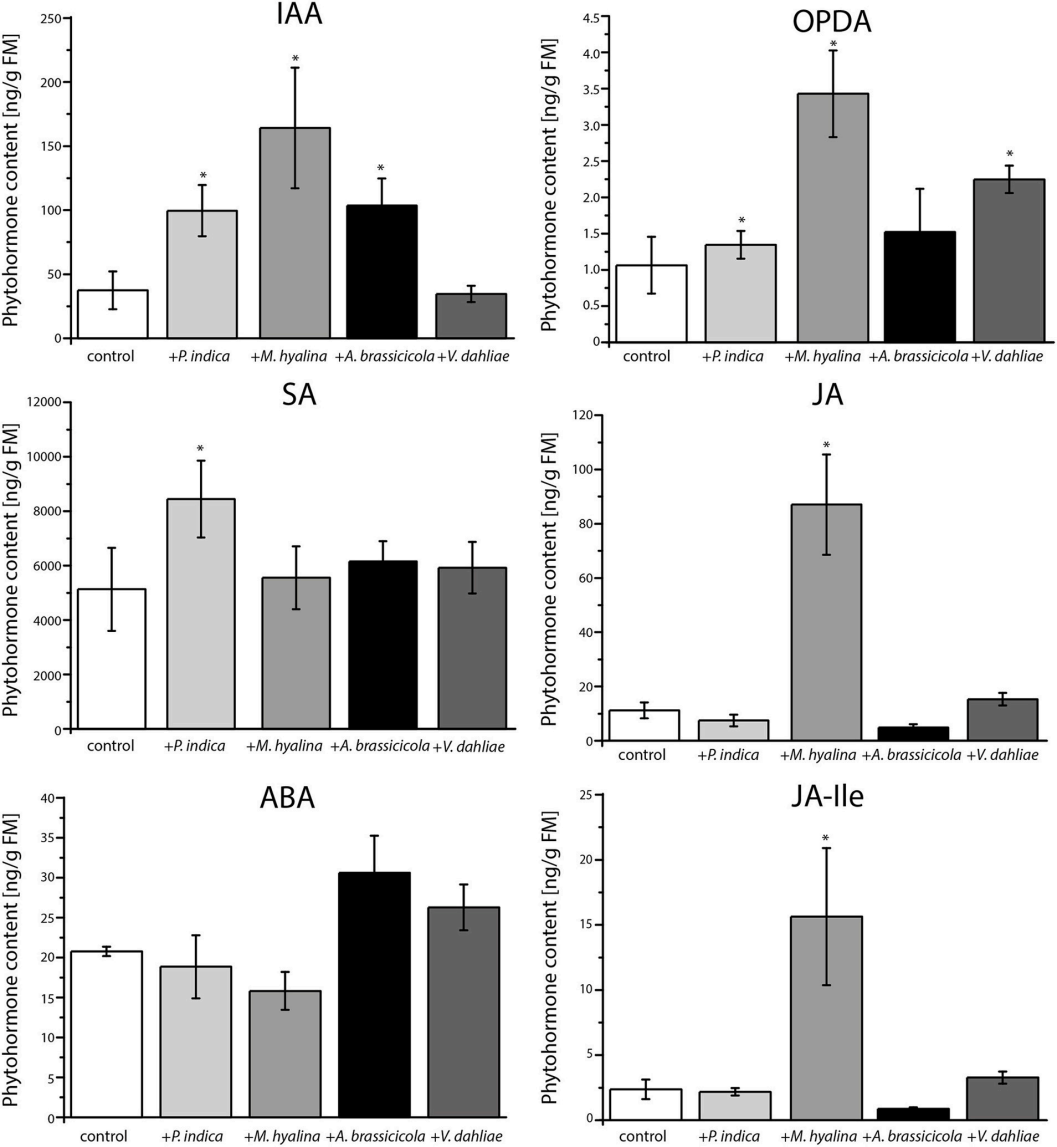
Phytohormone measurements of *A. thaliana* roots after one day of co-cultivation with *P. indica, M. hyalina, A. brassicicola*, and *V. dahliae*. (IAA, indole-3-acetic acid; ABA, abscisic acid; SA, salicylic acid; JA, jasmonic acid; OPDA, 12-oxophytodienoic acid (*cis* and *trans*); JA-Ile, jasmonoyl-isoleucine conjugate). Asterisks mark statistically significant differences against control treatment (*n* = 8; ^*^p < 0.05) as determined by one–way ANOVA followed by Tukey's test.

The phytohormone levels in Arabidopsis roots are strongly influenced by the 24 h incubation period with the different fungi (Figure 4). Compared to the uncolonized control, the IAA level in *P. indica-, M. hyalina-*, and *A. brassicicola-*colonized roots increased, whereas *V. dahliae* colonization had no effect. Interestingly, co-cultivation with *M. hyalina* resulted in the highest stimulation of the IAA level (~4-fold stimulation); *P. indica* and *A. brassicicola* showed a similar, but lower induction (~3-fold stimulation). These results are not consistent with expression data of the auxin reporter gene (Figures 2, 3).

However, quantification of the amounts of the defense-related phytohormones revealed that only *M. hyalina*, but not the other three fungi, stimulated the accumulation of the jasmonates JA and JA-Ile. In addition to *M. hyalina*, the JA precursor 12-oxophytodienoic acid (OPDA) showed a low, however significant increase after infection with *P. indica* and *V. dahliae*. The only other significant alteration induced by any of the fungi was a slight stimulation of the SA level in *P. indica*-infected roots. These results suggest the jasmonates play an important role during the early phase in the *M. hyalina*/Arabidopsis interaction, and that the sharp decline in the reporter gene expression in response to the two pathogens is probably not directly caused by the phytohormones but by factors influencing the cell fate in the symbiotic roots (cf. section Discussion).

### Phytohormone Levels in the Fungal Mycelia

**Figure 5:**
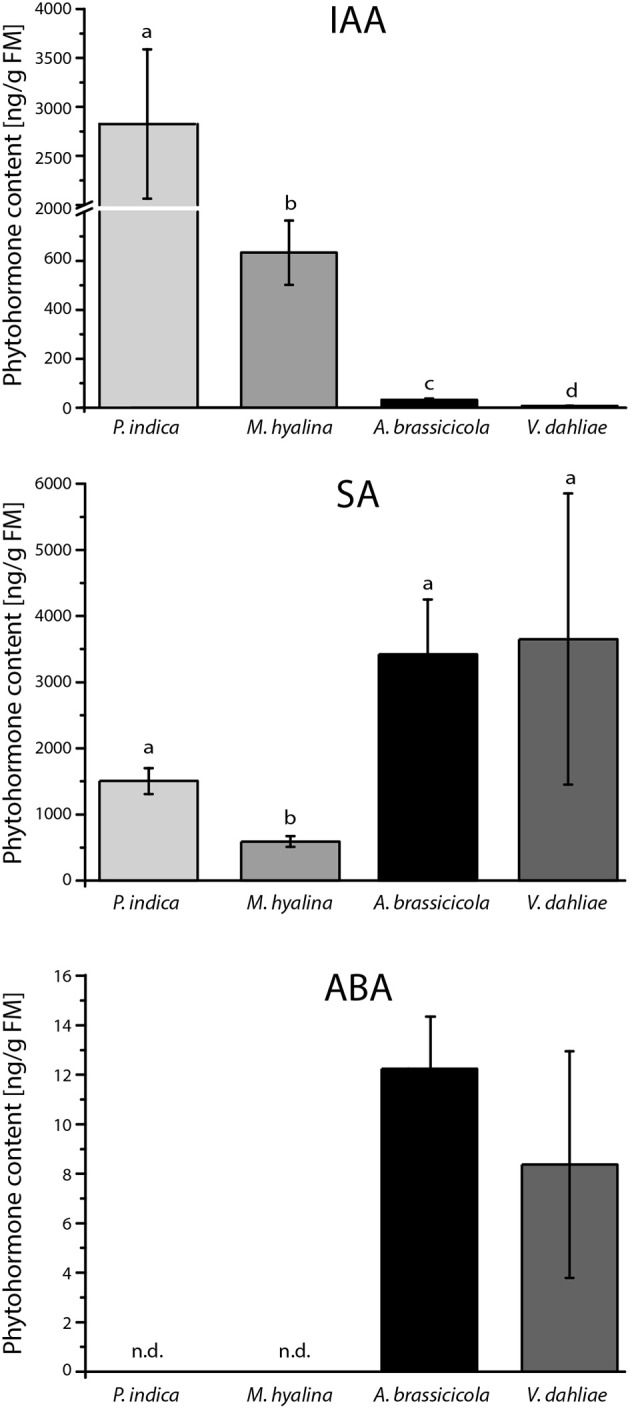
Phytohormone content of mycelia from *P. indica* cultivated on Kaefer's medium for three weeks and *M. hyalina, A. brassicicola*, and *V. dahliae* grown for 2 weeks on potato dextrose agar medium (IAA, indole-3-acetic acid; ABA, abscisic acid; SA, salicylic acid). Statistically significant differences between all fungal species were analyzed using one–way ANOVA. Different letters indicate significant differences among groups for *p* < 0.05, determined by Tukey's test (*n* = 3).

To investigate whether the observed differences in the regulation of the reporter gene in Arabidopsis roots are caused by differences in fungal auxin levels, the phytohormone concentrations were measured in the mycelia. Strikingly, the IAA contents in the four fungi varied tremendously. The highest level was found in *P. indica, M. hyalina* contained ~5 times less auxin, and the levels in the two pathogens was quite low (Figure 5). We also measured the amounts of the stress related JA, SA and abscisic acid. None of the mycelia contained JA and JA-Ile (data not shown). The lowest SA level was found in *M. hyalina*. *P. indica* contained twice as much and the pathogens 6–7 times more SA than *M. hyalina*. No abscisic acid was found in the two beneficial fungi, while the pathogens contained comparable levels (Figure 5). Taken together, the auxin levels that were detectable in the mycelia were consistent with the regulation of the auxin-responsive reporter gene in Arabidopsis roots.

### Jasmonate Impairs *DR5*::*EGFP-DR5v2*::*tdTomato* Fluorescence

**Figure 6:**
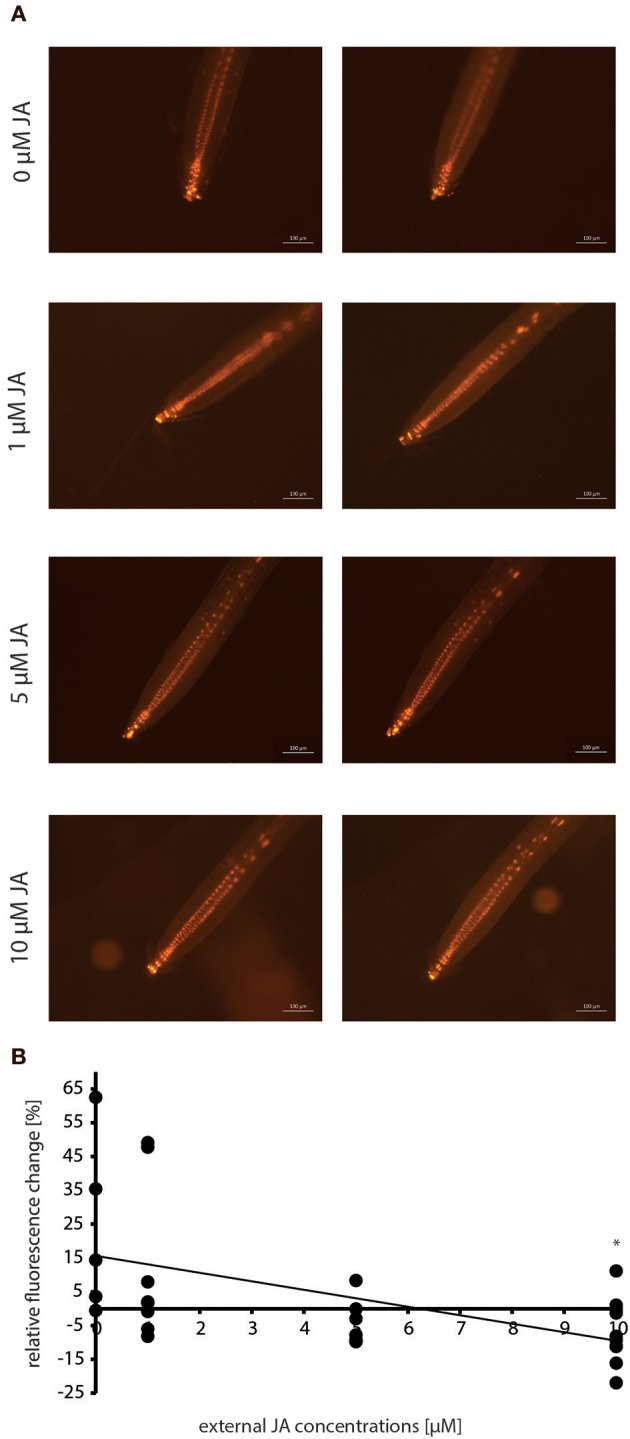
Dose-response curve of the reporter construct *DR5::EGFP-DR5v2::tdTomato* in response to incubation with 0, 1, 5, and 10 μM JA (jasmonic acid) before and after 24 h. **(A)** Fluorescence images of the tdTomato signal after 0 and 24 h. **(B)** Graphic presentation of the relative fluorescence change in response to exogenous JA concentrations (*n* = 6–10). Asterisks indicate significant differences to 0 μM JA (*p* < 0.05).

Due to the elevated jasmonate contents found during the *M. hyalina*/Arabidopsis interaction (Figure 4), the question arose whether jasmonates can affect the expression of the auxin reporter gene. Therefore, roots of 12 day-old *DR5*::*EGFP-DR5v2*::*tdTomato* seedlings were incubated with increasing JA concentrations for 24 h and the fluorescence was monitored. Incubation with 1 and 5 μM JA resulted in a stable but decreased tdTomato fluorescence intensity compared to control samples (Figures 6A,B). Application of 10 μM JA showed the significantly highest detectable loss of fluorescence (~20%) in the roots, supporting the hypothesis that increased JA levels during early *M. hyalina*/Arabidopsis interaction impede the activation of the IAA-responsive reporter construct.

### *P. indica* Induces the Formation of Lateral Root Primordia

**Figure 7:**
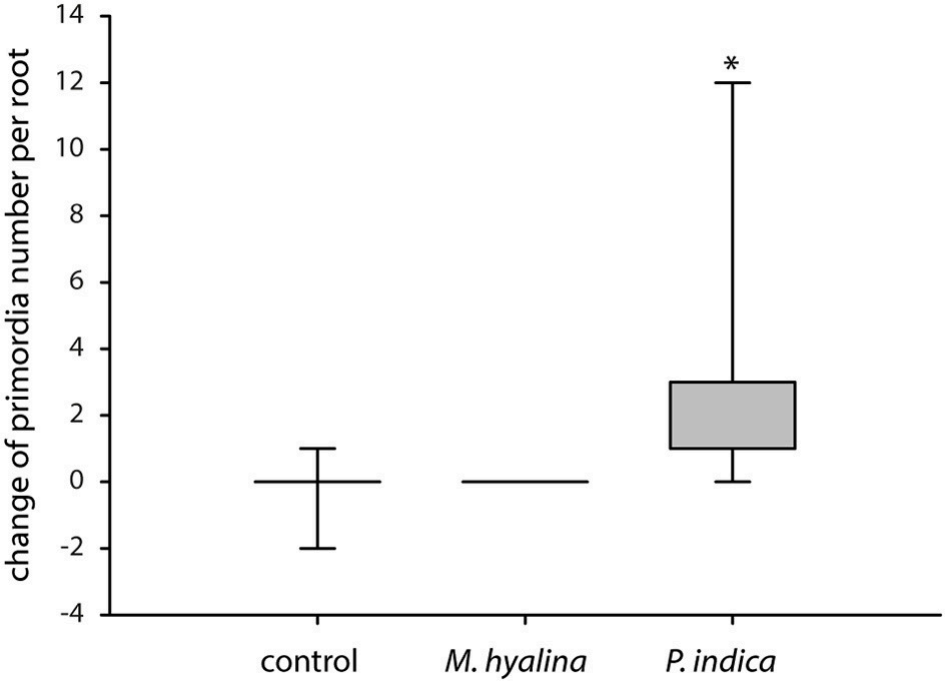
Measurement of lateral root primordia induction in *A. thaliana* after 1 day of co-cultivation with *M. hyalina* and *P. indica*. Graphical illustration of the absolute change of primordia number. The asterisk mark statistical significant differences against control and *M. hyalina* treatment (*n* = 5–7; ^*^p < 0.05).

The former results suggest that only *P. indica* can initiate processes which may result in the promotion of root development within the 24 h of experimentation. In accordance with this idea, we found that the initiation of lateral root primordia was stimulated by *P. indica* but not by *M. hyalina* (Figure 7).

## Discussion

### Expression of Auxin Reporter Is Dose-Dependent in Arabidopsis Roots

Liao et al. (2015) demonstrated that the auxin reporter construct *DR5*::*EGFP-DR5v2*::*tdTomato* induced a dose- and time-dependent fluorescence development as well as IAA-induced gene expression when Arabidopsis plants are treated with 0.0001–1.0 μM IAA. The construct was used to monitor the auxin level in different tissues of transformed *A. thaliana, P. tremula* × *alba, H. vulgare*, and *N. benthamiana* plants (Hilbert et al., 2012; Chen et al., 2013; Liao et al., 2015; Kirbis, 2017). A previous study showed that the EGFP signal was relatively low compared to the tdTomato signal (Kirbis, 2017). We observed the same for fungus-exposed and control Arabidopsis roots but did not observe differences in the expression for the two reporters (Figure 2A). Due to better resolution, we used the tdTomato fluorescence (Figures 2A,B) for our study and demonstrate that the expression is dose-dependent and shows expression in tissues and cells which are known to be involved in root growth (Figures 2B,C). With an increase in the applied IAA concentration, the fluorescence appeared also in tissues which are located shootwards of the elongation zone in the roots. The expression pattern matches known polar auxin transport mechanisms where IAA is translocated to the root apex and toward the root–shoot junction (Bennett et al., 1998; Reed et al., 1998; Rashotte et al., 2000; Casimiro et al., 2001). Therefore, the construct was used to monitor rapid changes in the auxin distribution in Arabidopsis roots in response to different root-interacting fungi.

### *P. indica*, but Not *M. hyalina* and the Pathogenic Fungi Activate the *DR5v2* Promoter in Arabidopsis Roots

The endophytic fungus *P. indica* promotes the growth of *A. thaliana* by enhancing both root and shoot biomass production (Peškan-Berghöfer et al., 2004; Camehl et al., 2011; Lee et al., 2011; Das et al., 2012). In Chinese cabbage it was demonstrated that colonization by *P. indica* also strongly enhances lateral root development (Lee et al., 2011; Dong et al., 2013). It is known that auxin plays a role in the beneficial interaction and that the fungus produces auxin and auxin precursors which interfere with hormones inside the plant (Sirrenberg et al., 2007; Vadassery et al., 2008; Hilbert et al., 2012; Xu et al., 2018). The auxin reporter was utilized to analyze the auxin kinetic in the early infection stages of *P. indica*-colonized roots as well as in roots colonized by *M. hyalina, A. brassicicola* and *V. dahliae* (Figure 3). Colonization with *P. indica* showed a strong, 10-fold increase in fluorescence in the entire root while the expression at the root tip stayed at a rather constant level (Figure 3B). Already 3 h after co-culture, the increase in the fluorescence (Figure 3A) indicates a higher amount of available IAA in the root tissue, which may be provided by the fungus or is produced by the plant. A similar (but only 3-fold) elevation of free IAA was observed in barley roots co-cultured with *P. indica* for 3 d (Hilbert et al., 2012). At later time points (5 and 14 days after infection) this effect was gone in barley which was also observed for Arabidopsis seedlings where the IAA level in colonized seedlings was not different from uncolonized controls 7, 10 and 14 days after infection (Vadassery et al., 2008; Hilbert et al., 2012). Our data suggests that *P. indica* induces a rapid increase in auxin levels during early recognition phases which might be crucial for reprogramming root development. Initiation of the formation of lateral root primordia can already be detected 24 h after co-cultivation with *P. indica*, but not with *M. hyalina* (Figure 7).

Phytohormones controlling the development of colonized roots can be of fungal or plant origin. Hilbert et al. (2012) showed that indole derivative production by *P. indica* is not required for growth promotion but for biotrophic colonization of barley roots. In other symbiotic interactions, promotion of root or plant growth was proposed to be caused by microbial phytohormones—in particular auxin (e.g., Contreras-Cornejo et al., 2009; Dudeja et al., 2012; Khan et al., 2014; Lee et al., 2015; Liao et al., 2017, and references therein). The examples demonstrate that fungal auxin or metabolites which can be converted to auxin in the plant, participate in growth regulatory effects in the host. Apparently, phytohormones that are active in the roots can be of either plant or microbial origin. Which one of the two sources might play a more important role in the symbiotic interaction and how these processes are related to circuits, which manipulate plant's phytohormone metabolism and function is probably symbiosis-specific.

*M. hyalina* did not induce such an auxin increase in the entire root during the first 24 h of co-culture, in fact, the level even decreased slightly about 20% (Figure 3B). Five h after co-cultivation, the fluorescence at the root tip increased by 30% and decreased again afterwards to half of the initial level. The initial increase in fluorescence during the recognition of the fungus by the plant could be caused by fungus-derived auxin which is later on stopped by other processes (cf. below). These results match the phenotype observed for *M. hyalina*-colonized Arabidopsis seedlings: we did not observe any significant increase in the root biomass while the aerial parts of the colonized seedlings became bigger (Johnson et al., 2018b).

In contrast, the fluorescence in the entire root decreased in response to the two pathogenic fungi *V. dahliae* and *A. brassicicola* after short periods of co-cultivation (Figure 3A). For *V. dahliae*, this was unexpected since it is well known that its interaction starts with a biotrophic phase associated with the stimulation of growth which is followed by a necrotrophic phase later on (Schnathorst, 1981; Reusche et al., 2014; Cho, 2015). In the *A. brassicicola* co-culture, the fluorescence was no longer detectable after 3 h while *V. dahliae*-infected plants lost the fluorescence ~6 h after co-cultivation (Figure 3B). This is accompanied by visible destruction of plant tissue and the root tip (data not shown). Both pathogens colonized the roots rapidly. For *V. dahliae*, the roots were covered with conidia already 6 h after infection (Zhao et al., 2014). Downregulation of the auxin response is likely caused by toxic effects: *V. dahliae* produces different phytotoxins and other molecules that induce the hosts' cell death associated with the degradation of host tissue (Fradin and Thomma, 2006). *A. brassicicola* produces the AB-toxin which is released by the germinating spores on host tissue (Oka et al., 2005).

### *M. hyalina* Induces Jasmonate and *P. indica* SA Accumulation in the Host Roots

Arabidopsis plants respond to colonization by different fungi with the accumulation of different phytohormones. Beneficial fungi like *P. indica* often induce SA, while necrotrophic fungi induce jasmonates (Schäfer et al., 2009; Sun et al., 2014; Johnson et al., 2018b; Scholz et al., 2018; Vahabi et al., 2018; Xu et al., 2018). To analyze whether the observed IAA-induced fluorescence changes during root colonization (Figure 3) are exclusively mediated by auxin changes or whether other phytohormones might also be involved, we measured the phytohormone profiles in the roots (Figure 4). Compared to uncolonized control roots, both beneficial fungi showed a significant accumulation of IAA within the first 24 h of co-culture. For later time points, quite different results for IAA accumulation were reported for *P. indica*-colonized roots (Vadassery et al., 2008; Lee et al., 2011; Hilbert et al., 2012; Hua et al., 2017). *M. elongata*-colonized maize roots and *M. alpina*-colonized *Crocus sativus* L. plants also showed significantly elevated IAA contents (Wani et al., 2017; Li et al., 2018). However, expression of auxin-responsive genes has never been investigated at the early time points studied here (Figure 3). Apparently, although *P. indica* and *M. hyalina* colonization results in the accumulation of auxin, the outcome is quite different. While *P. indica* induces fluorescence, *M. hyalina* does not. This suggests that auxin in the *M. hyalina*-colonized roots cannot (fully) activate the downstream IAA signaling cascade. A possible explanation could be that IAA signaling is antagonistically regulated by other hormones or elicitors.

Furthermore, *V. dahliae* did not induce IAA accumulation in Arabidopsis roots which matches the observed fluorescence. However, the IAA increase was similar for *A. brassicicola* and *P. indica*, although the pathogen did not induce the fluorescence from the auxin reporter (Figure 3). Similar observations were made in previous studies (Qi et al., 2012; Riet et al., 2016). This might be due to the rapidly induced cell death by the fast growing pathogen and its release of toxins. The importance of auxins for resistance against different pathogens has been repeatedly demonstrated: For instance, auxin biosynthesis defective mutants are more susceptible to *A. brassicicola* infection compared to wild-type plants (Bari and Jones, 2009; Kazan and Manners, 2009; Qi et al., 2012). Furthermore, *A. brassicicola* infection leads to the degradation of AUX/ IAA proteins indicating that the fungus activates the auxin signal transduction pathway (Qi et al., 2012). Since the hormone data in previous studies were measured for entire roots or even plants, it is difficult to compare them with locally occurring infections and generation of auxin maxima.

Recent studies have demonstrated an antagonistic crosstalk between IAA and SA, which in turn regulates plants' resistance to different fungi. It has been hypothesized that downregulation of auxin signaling is part of the SA-mediated disease-resistance mechanism (Navarro et al., 2006; Chen et al., 2007; Wang et al., 2007; Kazan and Manners, 2009). Our analysis of the SA content of colonized roots is consistent with this assumption, since roots with high IAA content did not show a strong accumulation of SA upon fungal infection at least with *A. brassicicola* and *M. hyalina* (Figure 4). However, *P. indica*-colonized roots showed a small increase in both—IAA and SA levels; the latter was also observed in our previous studies (Sun et al., 2014; Vahabi et al., 2018). This seemingly contradiction was addressed in a previous study showing that SA does not directly affect the IAA concentration but the auxin-dependent signaling (Wang et al., 2007). Colonization by *M. hyalina* did not result in elevated SA levels as already shown by Johnson et al. (2018b). The two pathogenic fungi did not induce SA. Similar results were obtained in previous studies where SA content was not elevated and SA mutants did not show a different susceptibility (Botanga et al., 2012; Sun et al., 2014; Scholz et al., 2018). Apparently, significant amounts of SA accumulate only during early phases of the *P. indica*/Arabidopsis interaction.

The class of jasmonates is mainly induced by wounding of plant tissue and plays a major role in defense against necrotrophic fungi (Thaler et al., 2004; Kachroo and Kachroo, 2009; Acosta and Farmera, 2010). Our study revealed that after 24 h of co-culture, the JA and JA-Ile contents were not significantly up-regulated in the roots colonized by the two pathogens (Figure 4). A previous study with *V. dahliae* showed the same result (Scholz et al., 2018), although a precursor of JA, OPDA, was stimulated by *V. dahliae*. In this study, we could not distinguish between the active *cis*- and inactive *trans*-OPDA, so a statement about the relevance of this JA precursor would not be appropriate. Interestingly, there was a clear difference in the induction of jasmonates between the two beneficial fungi. While *P. indica*-colonized roots contained the same levels of JA and JA-Ile as the uncolonized control roots, *M. hyalina* colonization resulted in a significant increase in the OPDA (3-fold), JA and JA-Ile levels (8-fold) (Figure 4). This appears to be restricted to the early recognition phase of the two partners, since we did not detect an increase of jasmonates in *M. hyalina*-colonized plants during later time points (Johnson et al., 2018b). A recent study in Arabidopsis indicated that JA interferes with auxin signaling independent of the JA-receptor complex COI1. The expression of auxin-inducible genes and lateral rooting is inhibited in Arabidopsis seedlings treated with JA (Ishimaru et al., 2018). These findings are supported by our experiment showing that the incubation with increasing concentrations of JA impairs the expression of the IAA-responsive reporter construct (Figures 6A,B) in spite of the presence of elevated IAA levels in *M. hyalina* colonized roots (Figure 4). Finally, although ABA is involved in plant defense (Figure 4), we did not find a significant accumulation of this hormone during the early phase of interaction for any of the four fungi with Arabidopsis roots.

The crosstalk of phytohormones during recognition and early phases of plant/fungus interaction is not well understood and most studies focus on defense-related hormones. The presented results and measuring techniques open new research fields, which will shine more light on the first steps in symbiotic interactions.

## Data Availability

All datasets generated for this study are included in the manuscript and/or the supplementary files.

## Author Contributions

AKM, AF, SS, and RO designed the experiments. AKM, AF, SÖ, and MA-T performed the experiments. AKM, AF, and MA-T analyzed the data. VG and BH provided and assisted with the light sheet microscope. AK, TL, and GT generated and provided the transgenic seed material. AKM, AF, SS, AM, and RO wrote the manuscript with contributions from all authors.

### Conflict of Interest Statement

The authors declare that the research was conducted in the absence of any commercial or financial relationships that could be construed as a potential conflict of interest.

## Supplementary Material

Supplementary Video S1 Visualization of auxin maxima using the reporter construct *DR5::EGFP-DR5v2::tdTomato* during plant-fungus interaction. Light sheet videos of 17 h-recordings of control without addition of fungal spores.

Supplementary Video S2 Visualization of auxin maxima using the reporter construct *DR5::EGFP-DR5v2::tdTomato* during plant-fungus interaction. Light sheet video of 17 h-recording of co-cultivation with *P. indica* spore solution.

Supplementary Video S3 Visualization of auxin maxima using the reporter construct *DR5::EGFP-DR5v2::tdTomato* during plant-fungus interaction. Light sheet video of 17 h-recording of co-cultivation with *M. hyalina* spore solution. Background fluorescence in *M. hyalina* co-culture is due to autofluorescence of spores.

Supplementary Video S4 Visualization of auxin maxima using the reporter construct *DR5::EGFP-DR5v2::tdTomato* during plant-fungus interaction. Light sheet video of 17 h-recording of co-cultivation with *A. brassicicola* spore solution.

Supplementary Video S5 Visualization of auxin maxima using the reporter construct *DR5::EGFP-DR5v2::tdTomato* during plant-fungus interaction. Light sheet video of 17 h-recording of co-cultivation with *V. dahliae* spore solution.
